# Association of NQO1 polymorphism with spontaneous breast cancer in two independent populations

**DOI:** 10.1038/sj.bjc.6601779

**Published:** 2004-04-27

**Authors:** H-J Menzel, J Sarmanova, P Soucek, R Berberich, K Grünewald, M Haun, H-G Kraft

**Affiliations:** 1 Inst. f. med. Biology and Human Genetics, University Innsbruck, Schopfstrasse 41, A-6020 Innsbruck, Austria; 2 National Institute of Public Health, Srobarova 48, Praha 10, 10042 Czech Republic; 3 Clinic of Internal Medicine, University Innsbruck, A-6020 Innsbruck, Austria

**Keywords:** breast cancer, SNP^5^, association study, NQO1, TP53

## Abstract

Eight different single-nucleotide polymorphisms (SNPs) in six different genes were investigated for possible association with breast cancer. We used a case–control study design in two Caucasian populations, one from Tyrol, Austria, and the other from Prague, Czech Republic. Two SNPs showed an association with breast cancer: R72P in*TP53* and P187S in *NQO1*. Six SNPs, Q356R and P871L in *BRCA1*, N372H in *BRCA2*, C112R (E4) and R158C (E2) in *ApoE* and C825T in *GNB3,* did not show any sign of association. The P187S polymorphism in *NQO1* was associated with breast cancer in both populations from Tyrol and Prague with a higher risk for carriers of the 187S allele. Combining the results of the two populations, we observed a highly significant difference (*P*=0.0004) of genotype and allele frequencies (odds ratio (OR)=1.46; 95% confidence interval (CI) 1.16–1.85; *P*=0.001) and of the homozygote ratio (OR=3.8; 95% CI 1.73–8.34; *P*=0.0001). Combining the two ‘candidate’ SNPs (P187S and R72P) revealed an increased risk for breast cancer of double heterozygotes (P187S/R72P) of the *NQO1* and *TP53* genes (OR=1.88; 95% CI 1.13–3.15; *P*=0.011), suggesting a possible interaction of these two loci.

In women, breast cancer is the most common malignant disease in industrialised countries. About 5–10% are the so-called familial cases, which can be mainly attributed to deleterious mutations in *BRCA1* and *BRCA2* (Dunning *et al*, 2001; Nathanson and Weber, 2001), and also for the remaining majority of spontaneous breast cancer cases a strong genetic component has been postulated (Lichtenstein *et al*, 2000). Although finally having a strong impact, the responsible genetic markers may be common, low-penetrance genetic variants that modify susceptibility to breast cancer.

Potential candidates for these markers are single-nucleotide polymorphism (SNPs) that alter the sequence or the expression of a gene product (Lander and Schork, 1994; Cargill *et al*, 1999; Gray *et al*, 2000; Risch, 2000; Sunyaev *et al*, 2001). A large variety of SNPs have already been investigated for their association with breast cancer (Dunning *et al*, 2001; Nathanson and Weber, 2001). These were SNPs in DNA repair genes, steroid hormone metabolism genes and carcinogen metabolism genes (see also, Goode *et al*, 2002). We have chosen eight different SNPs from six genes. These were Q356R and P871L in *BRCA1*, N372 H in *BRCA2*, R72P in*TP53*, C112R (E4) and R158C (E2) in *ApoE*, P187S in *NQO1* and C825 T in *GNB3.*

*BRCA1* and *BRCA2* are the well-established susceptibility genes for familial breast cancer (Dunning *et al*, 1997, 2001; Healey *et al*, 2000; Nathanson and Weber, 2001; Goode *et al*, 2002), *TP53* is a gene involved in apoptosis (Dumont *et al*, 2003), *ApoE* influences lipid metabolism and cardiovascular disease (Menzel *et al*, 1983) and may be involved in tumour proliferation (Moysich *et al*, 2000; Zunarelli *et al*, 2000), *NQO1* is engaged in carcinogen metabolism (Nebert *et al*, 2002) and *GNB3* is part of a signal transduction pathway (Siffert *et al*, 1998).

Searching the literature, no association with breast cancer has been found for Q356R and P871L (*BRCA1*), although the authors claimed that being homozygous for 356R might protect against breast cancer (Dunning *et al*, 1997). In case of the 372H allele (*BRCA2*), an increased risk for developing breast cancer has been observed (Healey *et al*, 2000) together with an association with foetal survival. The 72P allele of the polymorphism in *TP53* was only weakly associated with an increased risk for breast cancer (Nathanson and Weber, 2001). No association of breast cancer was observed with the apolipoprotein E polymorphism (Moysich *et al*, 2000; Zunarelli *et al*, 2000). For the P187S polymorphism in *NQO1*, conflicting results were published (Siegelmann-Danieli and Buetow, 2002; Hamajima *et al*, 2002). The C825 T polymorphism in *GNB3* has not been investigated so far.

A central consideration with case–control studies are spurious results due to a large variety of reasons (Lander and Schork, 1994). It is therefore mandatory to repeat published studies in different populations, and also null results should be published to avoid bias (Hemminki and Shields, 2002). One of the reasons for spurious results is a general statistical problem due to multiple testing. This can either be accounted for by applying statistical correction methods (e.g. Bonferoni) or by investigating at least two different populations.

Here we present the results of repetitive SNP association studies in our case–control study and of a new one that was performed in two independent populations, one from Tyrol, Austria and the other from Prague, Czech Republic. In addition, we have analysed the concomitant effect of two polymorphisms in two different genes in order to mimic the situation *in vivo* where the different genes/gene products do not act as single entities but as members of an ‘orchestra’, as suggested by Risch (2000).

## MATERIALS AND METHODS

### Control and patient populations

#### Controls from Tyrol

The controls (400 women) were randomly drawn from a group of 13 000 apparently healthy blood donors from Tyrol. All came from the same geographical area as the patient group. The mean age of the control group was 39±12 years. The control persons were all anonymous, and only their age and gender were known. For the investigation of a possible association of the N372H polymorphism with gender, we randomly chose additional 600 women and 1600 men.

#### Controls from Prague

The control group enclosed 231 women from Prague with a mean age of 60±23 years. Controls were recruited from the staff of the National Institute of Public Health, nurses and patients of collaborating hospitals in Prague and inhabitants of houses for elderly citizens living in the same urban area as the patients. Controls were interviewed and only those having no personal history neither of breast cancer nor other malignancies were included into the study. The composition of the control group was comparable to cases in terms of age. Controls were asked to read and sign an Informed Consent protocol.

#### Patient groups

The patient group from Tyrol, 220 women, had a mean age of 56±13 years and the patient group from Prague consisted of 190 women with an average age of 58±13 years. All patients gave Informed Consent. In all cases, the diagnosis of breast cancer was confirmed histological. The cases from Prague were all incident cases, whereas the cases from Tyrol were a mixture of incident and prevalent cases with a median of one survival year (mean 2.5±3.7).

#### Genotyping

All samples were genotyped by the 5′exonuclease assay with fluorescent MGB-probes on an ABI PRISM 7000 Sequence Detection System™ from Applied Biosystems. In addition, some samples were also genotyped by conventional methods (PCR and digestion) (Q356R, patients and controls from Tyrol and P187S, patients and controls from Prague) and by Pyrosequencing™ (P871L, controls from Tyrol). All methods gave identical results and not a single deviation was observed. The sequences of the primers designed for the analysis are given in appendix.

#### Statistical analysis

The *χ*^2^-test was used to compare the distribution of genotypes between cases, controls and expected genotypes assuming a Hardy–Weinberg equilibrium. The risk attributed to individual alleles or genotypes for breast cancer was calculated as odds ratio from 2 × 2 tables. A possible association of genotypes with age and survival was analysed by the Kruskal–Wallis test.

## RESULTS

The genotype frequencies of the eight SNPs investigated in the control and case groups from Tyrol and Prague are given in Tables 1

**Table 1:** Tyrol

**Gene**	**SNP**	**Controls**				**Patients**				**Statistics spec. Genotype**		**Statistics allelefr**
		**Rare allele frequency**	**Genotypes**	***N***	**(%)**	**Rare allele frequency**	**Genotypes**	***N***	**(%)**	**OR**	***P***	**OR**
												**95% CI**
												***P***
*BRCA1*	Q356R	0.09	QQ	335	(84)	0.09	QQ	189	(88)	1.0		0.76
			QR	58	(15)		QR	25	(11)	0.76	0.29	0.47–1.22
			RR	5	(1)		RR	2	(1)	0.71	0.68	0.24
*BRCA1*	P871L	0.30	PP	184	(48)	0.33	PP	91	(43)	1.0		1.14
			PL	171	(45)		PL	102	(49)	1.11	0.57	0.88–1.49
			LL	29	(8)		LL	18	(8)	1.20	0.59	0.3
*BRCA2*	N372H	0.27	NN	482	(53)	0.29	NN	104	(50)	1.0		1.08
			NH	361	(40)		NH	91	(43)	1.17	0.33	0.85–1.37
			HH	69	(7)		HH	16	(7)	1.07	0.80	0.5
*TP53*	R72P	0.24	RR	191	(59)	0.28	RR	109	(53)	1.0		1.25
			RP	112	(34)		RP	79	(38)	1.24	0.26	0.93–1.67
			PP	22	(7)		PP	19	(9)	1.51	0.21	0.16
*GNB3*	C825T	0.33	CC	176	(47)	0.33	CC	102	(48)	1.0		1.11
			CT	159	(43)		CT	82	(38)	0.91	0.60	0.86–1.45
			TT	36	(10)		TT	31	(14)	1.49	0.15	0.4
*ApoE*	C112R (E4)	0.13	CC	292	(77)	0.15	CC	159	(73)	1.0		1.12
			CR	81	(21)		CR	52	(24)	1.18	0.42	0.78–1.60
			RR	9	(2)		RR	6	(3)	1.22	0.71	0.5
*ApoE*	R158C (E2)	0.08	RR	288	(85)	0.07	RR	185	(86)	1.0		0.91
			RC	49	(14)		RC	31	(14)	0.98	0.95	0.55–1.46
			CC	2	(1)		CC	0	(0)	—		0.7
NQO1	P187S	0.17	PP	290	(67)	0.21	PP	133	(61)	1.0		**1.37**
			PS	126	(31)		PS	76	(35)	1.32	0.13	**1.01–1.85**
			SS	8	(2)		SS	9	(4)	2.45	0.06	**0.035**
OR=odds ratio; CI=confidence interval; P=probability that the difference is caused by chance.												

2

**Table 2:** Prague

**Gene**	**SNP**	**Controls**				**Patients**				**Statistics spec. genotype**		**Statistics allelefr**
		**Rare allele frequency**	**Genotypes**	***N***	**(%)**	**Rare allele frequency**	**Genotypes**	***N***	**(%)**	**OR**	***P***	**OR**
												**95% CI**
												***P***
*BRCA1*	Q356R	0.06	QQ	128	(87)	0.08	QQ	83	(84)	1.0		1.27
			QR	19	(13)		QR	16	(16)	1.30	0.48	0.60–2.67
			RR	0	(0)		RR	0	(0)	—	—	0.5
*BRCA1*	P871L	0.36	PP	64	(43)	0.32	PP	44	(45)	1.0		0.85
			PL	63	(42)		PL	45	(46)	1.04	0.89	0.57–1.26
			LL	22	(15)		LL	9	(9)	0.60	0.24	0.4
*BRCA2*	N372H	0.26	NN	84	(55)	0.26	NN	53	(57)	1.0		0.93
			NH	57	(38)		NH	35	(37)	0.97	0.92	0.61–1.47
			HH	11	(7)		HH	6	(6)	0.86	0.79	0.8
*TP53*	R72P	0.25	RR	84	(56)	0.30	RR	49	(51)	1.0		1.31
			RP	58	(39)		RP	35	(37)	1.03	0.90	0.85–2.01
			PP	8	(5)		PP	11	(12)	2.36	0.08	0.2
*GNB3*	C825T	0.30	CC	73	(48)	0.30	CC	48	(50)	1.0		1.00
			CT	66	(43)		CT	39	(41)	0.90	0.70	0.66–1.52
			TT	13	(8)		TT	9	(9)	1.05	0.91	1.0
*ApoE*	C112R (E4)	0.12	CC	115	(77)	0.09	CC	79	(84)	1.0		0.73
			CR	34	(22)		CR	13	(14)	0.56	0.10	0.38–1.39
			RR	1	(1)		RR	2	(2)	2.91	0.36	0.3
*ApoE*	R158C (E2)	0.09	RR	127	(84)	0.08	RR	84	(84)	1.0		0.89
			RC	23	(15)		RC	16	(16)	1.05	0.90	0.45–1.78
			CC	2	(1)		CC	0	(0)	—		0.7
NQO1	P187S	0.13	PP	175	(76)	0.20	PP	127	(67)	1.0		**1.76**
			PS	53	(23)		PS	48	(25)	1.25	0.34	**1.20–2.60**
			SS	3	(1)		SS	15	(8)	**6.89**	**0.006**	**0.002**
OR=odds ratio; CI=confidence interval; P=probability that the difference is caused by chance.												

, respectively. In every group or subgroup, the genotype frequencies were in accordance with the assumption of a Hardy–Weinberg equilibrium. No association between genotype frequencies and age was observed except for the C112R (E4) polymorphism in the controls from Prague. Also, no association between genotypes and survival was discovered in the patient group from Tyrol, which is in agreement with the paper of Goode *et al* (2002). When allele frequencies were compared between cases and controls, only one significant deviation was observed: the P187S SNP in the NQO1 gene (see Tables 1 and 2). The 187S allele was found significantly more frequently in breast cancer patients than in controls. The same deviation could be observed in both populations from Tyrol and Prague. Regarding all other SNPs no significant differences were observed (Tables 1 and 2).

With respect to this association, we also tested for possible deviations of genotype frequencies of the P187S SNP and compared the homozygote ratio between cases and controls. In the group from Prague, there were in addition to the significant allele frequency difference also highly significant differences of all genotypes (*P*=0.0025) and of the ratio of the two homozygous genotypes (PP/SS) (odds ratio (OR)=6.9; 95% confidence interval (CI) 1.8–30.6; *P*=0.0006).

Between the respective groups from Tyrol and Prague, there was no significant difference of the allele frequencies and genotype frequencies of all SNPs under investigation. In order to increase power, we therefore combined the respective cases and controls groups from the two middle European populations. Again, in case of the P187S SNP, there was a significant difference of the allele frequencies (OR=1.46; 95% CI 1.16–1.85; *P*=0.001), the genotype frequencies (*P*=0.0003) and the homozygote ratio between patients and controls (OR=3.8; 95% CI 1.73–8.34; *P*=0.0001). In addition, also the R72P SNP in the *TP53* gene showed a borderline significant difference regarding allele frequencies (OR=1.27; 95% CI 1.00–1.61; *P*=0.044) and the homozygote ratio (OR=1.77; 95% CI 1.00–3.13; *P*=0.04). No significant difference was observed between cases and controls when we compared frequencies of heterozygotes, both P187S and R72P, and of homozygotes for the common allele, P187 and R72, respectively.

We also analysed the H372 H polymorphism in the *BRCA2* gene to test for a previously found association with gender (Healey *et al*, 2000) in 2442 controls (1530 men and 912 women). No difference of genotype frequencies between men and women (*P*=0.73) was found and also no deviation from expected frequencies assuming Hardy–Weinberg equilibrium (*P*=0.99).

Since we had analysed eight different SNPs in six different genes, we also investigated for the presence of linkage disequilibrium and for pair-wise locus association. The two SNPs of *BRCA1* and *ApoE*, respectively, were in linkage disequilibrium as expected.

The calculation of association for pair-wise loci as outlined by the two-locus genetic model of Risch (2000) was performed for the polymorphism at the *NQO1* and *TP53* gene loci, since these were the only two polymorphic sites that showed a significant association if the two studies were combined. Further, the associations of the two gene loci with breast cancer are the only ones that showed in both studies a trend in the same direction and a gene dosage effect (Hemminki and Shields, 2002) in contrast to the other polymorphic sites. When single-locus heterozygote and common allele homozygote frequencies were compared between patients and controls, there was no significant difference. The double heterozygotes (P187S/R72P) were more frequent in patients (40%) than in controls (28%) compared to the common allele double homozygotes (P187/R72) (67 *vs* 71%; OR=1.88; 95% CI 1.12–3.15; *P*=0.011).

Individuals with two or more “deleterious” alleles (Table 3

**Table 3:** Genotype combinations at the *NQO1* and *TP53* loci for the combined populations from Tyrol and Prague

		**Controls**		**Patients**		**Δ% controls−patients**	**Statistics specific genotype^b^**	
**Genotype NQO1/TP53**	**Number of “deleterious”^a^ alleles in genotype**	***n***	**%**	***n***	**%**		**OR**	**P**
SS/PP	4	1	0.22	1	0.34	−0.12	1.79	0.68
								
SS/rP	3	14	3.2	14	4.7	−1.5	1.79	0.14
pS/PP								
								
SS/rr								
pp/PP	2	64	14.4	72	24.3	−9.9	2.02	0.0009
pS/rP								
								
pS/rr	1	184	41.4	104	35.1	6.3	1.01	0.94
pp/rP								
								
pp/rr	0	181	40.8	101	34.1	6.7	1.0	
								
Total		444	296					
^a^ deleterious alleles are 187S at the *NQO1* locus and 72P at the *TP53* locus; “non deleterious” alleles are 187p at the *NQO1* locus and 72r at the *TP53* locus.								
^b^ OR=odds ratio; *P*=probability that the difference is caused by chance.								

) were more frequent in the patients group than in the control group (OR=1.97; 95% CI 1.31–2.97; *P*=0.0006). On the other hand, those with only one deleterious allele or those with no deleterious allele at all (Table 3) had comparable ratios in both patients and controls (OR=1.03; 95% CI 0.75–1.53; *P*=0.7).

## DISCUSSION

This association study of breast cancer patients analysing eight different SNPs in two different populations has shown in part accordance with previously published papers, in part divergence with published results and finally new results. The investigated SNPs in *BRCA1* and *BRCA2* showed no association with breast cancer in the two populations from Austria and the Czech Republic. This is in agreement with previous results for the P871L polymorphism (Dunning *et al*, 1997), but there are discrepancies for the Q356R polymorphism in *BRCA1* (Dunning *et al*, 1997) and the N372H polymorphism in *BRCA2* (Healey *et al*, 2000). One paper (Dunning *et al*, 1997) claimed a protective effect for the R356 allele in the homozygous state. In our population from Tyrol, the homozygotes were found in the same ratio in both the control and the patient groups. There were no homozygotes in the population from Prague, but because of the small group size this was still in accordance with Hardy–Weinberg equilibrium. It remains doubtful if the absence of the 356R homozygotes is real or spurious.

The association of the N372H polymorphism with breast cancer observed by Healey *et al* (2000) could not be confirmed in this study. It is, however, striking that their observation was only found in an English population but not in the German and Finnish population in the later publication. It remains unclear if this is a special condition in the English population. The other published results (Healey *et al*, 2000) concerning the deviation from Hardy–Weinberg equilibrium and the differences between men and women of the N372H polymorphism could not be confirmed by this study. When we tested for deviation from Hardy–Weinberg by a *χ*^2^-test using the published frequencies of the control groups, we obtained no significant deviation. Our own results gave an excellent conformity with Hardy–Weinberg equilibrium.

For the R72P polymorphism in the *TP53*-gene, only a weak association was observed and only in the combined population. In the literature, there are some publications stating the same result but with smaller group sizes (Sjalander *et al*, 1996; Wang-Gohrke *et al*, 1998). Most likely the effect is very small even in the homozygous state of the 72P allele.

The C825T polymorphism in GNB3 apparently does not influence the carcinogenesis of breast cancer, which is also true for the Apo E polymorphism. The latter is in agreement with the literature (Moysich *et al*, 2000; Zunarelli *et al*, 2000).

The association of the 187S allele of *NQO1* could be observed in both populations from Prague and Tyrol. The effect was more significant in the population from Prague than in the Tyrolean population, which might be explained by the different recruiting of the control groups. The Czech group are age-matched women, partly from a selected environment, with no evidence of breast cancer, whereas the women from Tyrol are blood donors between the age of 18 and 67 years incorporating a substantial number of women who might still get breast cancer in their later years.

Choosing the right control population is a very critical aspect in every case–control study. It has been a longstanding prerequisite that controls should be age matched to patients. Unfortunately, this might lead in the case of late-onset diseases like breast cancer to stratification due to selection bias. At the age in which most of the cases of breast cancer occur, also a lot of circulatory diseases occur that might change profoundly the composition of an age-matched control group. The fact that similar results have been gained, although different recruiting strategies have been used, further confirms that the actual findings of this study are real.

Comparison of our data on the P187S polymorphism in *NQO1* with those in the literature shows inconsistency (Hamajima *et al*, 2002; Siegelmann-Danieli and Buetow, 2002), particularly with the one from Japan where the frequency for the 187S homozygotes was 16.5% in the controls and 14.3% in the breast cancer patients group. In contrast, a group from Philadelphia (Dunning *et al*, 1997) observed no significant difference, but the frequency of the 187S allele was 19% in the case group and 15% in the control group, which is in the same range as in our groups and shows the same trend as this study. The fact that the American study (Siegelmann-Danieli and Buetow, 2002) showed no significant difference in *NQO1* polymorphism may be due to the low numbers of 187S homozygotes in the patient group and high numbers of 187S homozygotes in the control group that might have occurred by chance. Combining the numbers of the studies for this polymorphism from Tyrol, Prague and Philadelphia, very significant differences in allele frequency, genotypes and homozygotes ratio (data not shown) are observed.

NAD(P)H: quinone oxireductase (NQO1) is an enzyme that is involved in metabolising numerous endogenous and environmental quinones. Exchange of the Proline at position 187 by Serine leads to a nonfunctional enzyme (Nebert *et al*, 2002). Individuals homozygous for the 187S allele have a high risk for aplastic anaemia and leukaemia (Nebert *et al*, 2002). Reports about the association of the P187S polymorphism with lung cancer are inconsistent (Chen *et al*, 1999; Lewis *et al*, 2001). The association of the P187S polymorphism with breast cancer found in this study is the first reported in the literature and should be further investigated.

The increased risk of the double heterozygotes (P187S/R72P) to develop breast cancer is a new finding, which is similar to the observation that double heterozygotes for the Factor V “Leiden” and the Prothrombin mutation G20210>A have a 20-fold risk for developing thrombosis, whereas the risk for single heterozygotes is only five-fold and four-fold, respectively (Emmerich *et al*, 2001), and agrees with the two-locus genetic model of Risch (2000). Whereas the interactions of the *F5* and *F2* gene products in thrombosis are well understood, the way of interaction of the *NQO1* and *TP53* products in breast cancer can only be speculated. Hydroquinone, a substrate for detoxification by the NQO enzyme, can induce apoptosis (Moran *et al*, 1999), which is less stimulated by the 72P variant of p53 (Dumont *et al*, 2003). How these pathways are actually interwoven remains open for further investigations.

## Appendix

Primer for allele-specific PCR of Q356R:

BRCA1

Q356R-For:GACAGAATGAATGTAGAAAAGGCTGA

Q356R-Rev:ACGTCCAATACATCAGCTACTTTGG

356Q: GAGAAAAGAATGGAATAAGAA

356R: GAGAAAAGAATGGAATAAGAG

Blocker356Q: GAGAAAAGAATGGAATAAGAddG

Blocker356R: GAGAAAAGAATGGAATAAGAddA

Analysis was performed according to Orou *et al* (1995)

Primer for pyrosequencing of P871L:

BRCA1

P871L-For:TAACCACAGTCGGGAAACAAG

P871L-Rev:AACCACAGGAAAGCCTGCAGTG

Sequencing Primer:CCAGTCATTTGCTC

Primer for PCR and digest for P187S:

*NQO1*

P187S-For:TCCTCAGAGTGGCATTCTGC

P187S-Rev:TCTCCTCATCCTGTACCTCT

The PCR product was digested with *HinfI*

and the analysis was performed according to Lewis *et al* (2001)

Primer for 5′exonuclease assay:

BRCA1

Q356R-For:GAATGCTGATCCCCTGTGTGA

Q356R-Rev:AACATCTTCAGTATCTCTAGGATTCTCTGA

MGB

356Q: Fam-AATAAGCaGAAACTG

356R: Vic-TGGAATAAGCgGAAAC

BRCA1

P871L-For:AACTTGATGCTCAGTATTTGCAGAATA

P871L-Rev:TCCTCTTCTGCATTTCCTGGAT

MGB

871P: Fam-TTGCTCcGTTTTCAA

871L: Vic-ATTTGCTCtGTTTTCA

*BRCA2*

N372H-For:AACCAAATGATACTGATCCATTAGATTC

N372H-Rev:CAACTTCCTTGGAGATTTTGTCACT

MGB

372N: Fam-TGTAGCAaATCAGAAGC

372H: Vic-ATGTAGCAcATCAGAAG

TP53

R72P-For:TCCCCGGACGATATTGAACA

R72P-Rev:CCGCCGGTGTAGGAGCT

MGB

72R: Vic-CTGCTCCCCgCGTG

72P: Fam-CTGCTCCCCcCGTG

*GNB3*

C825T-For:TCCCACGAGAGCATCATCTG

C825T-Rev:TCGTCGTAGCCAGCGAATAGT

MGB

825C: Fam-CACGTCcTGTGGCC

825T: Vic-ATCACGTCtTGTGGCCT

*ApoE*

C112R-For: GCTGGGCGCGGACAT

C112R-Rev: CCTCGCCGCGGTACTG

MGB

112C: Vic-CCGCaCACGTCCT

112R: Fam-CGCtCACGTCCT

R158C-For: CCGCGATGCCGATGAC

R158C-Rev: GCCCCGGCCTGGTACA

MGB

158R: Fam-AGAAGcGCCTGGCA

158C: Vic-CAG AAG tGC CTG GCA

*NQO1*

P187S-For:TGCATTTCTGTGGCTTCCAA

P187S-Rev:CTGGAGTGTGCCCAATGCTA

MGB

187P: VIC-TCTTAGAAcCTCAACTGACA

187S: FAM-TCTTAGAAtCTCAACTGACA
